# Continued Androgen Signalling Inhibition improves Cabazitaxel Efficacy in Prostate Cancer

**DOI:** 10.1016/j.ebiom.2021.103681

**Published:** 2021-11-05

**Authors:** Lisanne Mout, Martin E. van Royen, Corrina de Ridder, Debra Stuurman, Wesley S. van de Geer, Rute Marques, Stefan A.J. Buck, Pim J. French, Harmen J.G. van de Werken, Ron H.J. Mathijssen, Ronald de Wit, Martijn P. Lolkema, Wytske M. van Weerden

**Affiliations:** a Department of Medical Oncology Erasmus MC Cancer Institute, Dr. Molewaterplein 40, 3015, GD, Rotterdam, the Netherlands; b Department of Urology Erasmus University MC, Dr. Molewaterplein 40, 3015, GD, Rotterdam, the Netherlands; c Department of Pathology Erasmus University MC, Dr. Molewaterplein 40, 3015, GD, Rotterdam, the Netherlands; d Cancer Treatment Screening Facility Erasmus University MC, Dr. Molewaterplein 40, 3015, GD, Rotterdam, the Netherlands; e Department of Neurology Erasmus University MC, Dr. Molewaterplein 40, 3015, GD, Rotterdam, the Netherlands; f Cancer Computational Biology Center Erasmus MC Cancer Institute, University Medical Center, Dr. Molewaterplein 40, 3015, GD, Rotterdam, the Netherlands

**Keywords:** Taxane chemotherapeutics, Androgen receptor, AR targeted inhibitors, castration resistant prostate cancer, combination treatments

## Abstract

**Background:**

**:** The androgen receptor (AR) pathway is a key driver of neoplastic behaviour in the different stages of metastatic prostate cancer (mPCa). Targeting the AR therefore remains the cornerstone for mPCa treatment. We have previously reported that activation of AR signalling affects taxane chemo-sensitivity in preclinical models of castration resistant PCa (CRPC). Here, we explored the anti-tumour efficacy of the AR targeted inhibitor enzalutamide combined with cabazitaxel.

**Methods:**

**:** We used the AR positive CRPC model PC346C-DCC-K to assess the *in vitro* and *in vivo* activity of combining enzalutamide with cabazitaxel. Subsequent validation studies were performed using an enzalutamide resistant VCaP model. To investigate the impact of AR signalling on cabazitaxel activity we used quantitative live-cell imaging of tubulin stabilization and apoptosis related nuclear fragmentation.

**Findings:**

**:** Enzalutamide strongly amplified cabazitaxel anti-tumour activity in the patient-derived xenograft models PC346C-DCC-K (median time to humane endpoint 77 versus 48 days, P<0.0001) and VCaP-Enza-B (median time to humane endpoint 80 versus 53 days, P<0.001). Although enzalutamide treatment by itself was ineffective in reducing tumour growth, it significantly suppressed AR signalling in PC346C-DCC-K tumours as shown by AR target gene expression. The addition of enzalutamide enhanced cabazitaxel induced apoptosis as shown by live-cell imaging (P<0.001).

**Interpretation:**

**:** Our study demonstrates that cabazitaxel efficacy can be improved by simultaneous blocking of AR signalling by enzalutamide, even if AR targeted treatment no longer affects tumour growth. These findings support clinical studies that combine AR targeted inhibitors with cabazitaxel in CRPC.

**Research in context**

**Evidence before this study**

Androgen receptor (AR) signalling remains a key driver of neoplastic behaviour and therapeutic resistance in castration resistant prostate cancer (CRPC). We have previously reported that AR signalling drives taxane resistance in models of CRPC.

**Added value of this study**

Here we show that concomitant targeting of AR signalling by enzalutamide improves cabazitaxel activity even in tumours that have developed enzalutamide resistance. Here enzalutamide suppressed AR signalling, although tumour growth was not impacted, which reflected the treatment resistant phenotype of the CRPC models. Targeting the AR by enzalutamide enhanced cabazitaxel induced apoptosis and further suppressed proliferation. Overall, our study shows that cabazitaxel treatment efficacy is strongly enhanced by targeting AR signalling in models of CRPC.

**Implications of all the available evidence**

Our study paves the way for combining taxane chemotherapeutics with AR targeted agents, even for CRPC patients that have progressed on AR targeted compounds.

Alt-text: Unlabelled box

## 1 Introduction

The androgen receptor (AR) pathway is a major driver of neoplastic behaviour in the different stages of metastatic prostate cancer (mPCa). Hence, androgen deprivation therapy (ADT), which blocks the production of the AR ligands testosterone and dihydrotestosterone levels, is the cornerstone of mPCa treatment. Although ADT effectively induces disease regression in the vast majority of patients, disease recurrence is inevitable and is classified as castration resistant prostate cancer (CRPC). Multiple studies have shown that the AR continues to play a major role in ADT resistance and progression to CRPC, as AR amplifications and mutations conferring androgen hypersensitivity and ligand promiscuity are frequently identified in samples from CRPC patients [1,2]. Large-scale whole genome sequencing studies showed that AR alterations occur in 80-85% of CRPC patients, which are rare in castration naïve patients [3,4]. This underlines the importance of continued suppression of AR signalling by ADT or targeted inhibitors such as enzalutamide in CRPC patients. We have previously reported on the impact of androgens and AR signalling on taxane treatment efficacy in CRPC. Taxanes represent a vital therapeutic option for CRPC, however, treatment efficacy is limited by intrinsic and acquired resistance [5]. We showed that testosterone supplementation strongly impairs the activity of both docetaxel and cabazitaxel *in vivo* [6,7]. Moreover, stimulating AR signalling by testosterone was able to counteract docetaxel induced long-term tumour regression, demonstrating that AR signalling directly contributed to taxane resistance. We therefore hypothesized that taxane treatment efficacy is enhanced by simultaneous blockade of AR signalling, through targeted inhibitors such as enzalutamide. Here, we show that combining enzalutamide with cabazitaxel, indeed significantly improved activity in enzalutamide resistant patient-derived xenograft (PDX) models. We demonstrate that targeting the AR enhances the rate of cabazitaxel-induced apoptosis. Our results support new combination treatments of taxane chemotherapeutics with AR targeted agents in CRPC patients, even for patients that have progressed on the same AR targeted agent.

## 2 Methods

### 2.1 Cell culture and treatment sensitivity *in vitro*

The AR expressing CRPC model PC346C-DCC-K was obtained by long-term exposure to steroid stripped cell culture conditions as described previously [6,8]. VCaP-Enza-B was obtained through long-term propagation of the parental VCaP cell line (RRID:CVCL_2235, a kind gift from Dr. Pienta, Baltimore, Maryland, USA) in RPMI (cat. no. BE12-167F, Lonza, Basel, Switzerland) supplemented with 10% FCS and 10 µM enzalutamide (cat. no 1613, Axon Medchem, Groningen, the Netherlands). Cell line authentication was performed by short tandem repeat analysis using the Promega PowerPlex 16 kit and compared to the parental cell lines. Absence of mycoplasma contamination was assessed regularly (MycoAlert kit, cat. no. LT07-318, Lonza). For cell viability assays, PC346C-DCC-K and VCaP-Enza-B cells were plated at a cell density of 5*10^3^ cells per well in 100 µl medium. VCaP-Enza-B cells were maintained in RPMI containing 10% steroid stripped (DCC) serum, while PC346C-DCC-K cells were maintained in PGM-DCC medium [8,9]. The following day, cells were exposed to a dose-range of cabazitaxel (0-10 nM, Sanofi-Aventis, Paris, France), enzalutamide or the combination of cabazitaxel with 1 µM enzalutamide in the presence of 0•1 nM R1881 (cat. no. D5027, Sigma-Aldrich, Saint-Louis, MO). After 7 (VCaP-Enza-B) or 10 days (PC346C-DCC-K), cell viability was measured by the MTT assay (cat. no. M2128, Sigma-Aldrich) as described previously, and normalized to vehicle control [9]. Characterization of the newly acquired VCaP-Enza-B cell line was performed by examining the RNA and protein expression of the *AR, AR-V7* and several *AR* target genes. In short, RNA was isolated using the RNeasy mini kit (#74104, Qiagen) and qRT-PCR was performed as described previously [6]. Gene-expression of the following targets was assessed using TaqMan assays; *AR, AR-V7, KLK3* (PSA) (using custom assays), *FKBP5* and intact *TMPRSS2* (using commercial kits Hs01561006 and Hs01120965 respectively, ThermoFisher Scientific). *PBGD* and *HPRT1* were measured by SYBER Green assay (SensiMix Syber Lo-ROX). The custom TaqMan assays and primer sequences used for SYBER Green have been described previously [6,10]. For protein expression, 20 µg protein lysate was used for immunoblotting and blots incubated with the following primary antibodies; rabbit-anti-AR targeting the N-terminal domain (Sp107, #200R-14, Cell Marque at 1:2000), anti-PSA (#A0562, Dako Agilent at 1:1000) and anti-β-actin used as loading control (#A1978, Sigma-Aldrich, RRID:AB_476692 at 1:10000).

## 3 Ethics

All animal experiments were approved by the Animal experiment committee under the Dutch experiments on Animal Act, with the reference number AVD101002017867. The current study is in compliance with the Arrive guidelines. Group size and experimental set-up were based on pilot experiments or previous studies using taxane treatment *in vivo* [7]. All operations (tumour inoculation, blood sampling) were conducted under adequate anaesthesia to minimize animal discomfort as described previously [6]. Subcutaneously growing tumours only cause mild discomfort.

### 3.1 Combining enzalutamide with cabazitaxel *in vivo*

Fifty-two, six weeks old, NMRI nu/nu male mice (Janvier, Le Genest Saint Isle, France) were subcutaneously inoculated with 5*10^6^ PC346C-DCC-K cells. Tumour growth was observed after 2-5 weeks in 41 (∼80%) mice, to adhere to the predefined group size we added five mice from the RNA-sequencing experiment described below, as they were inoculated on the same day. Once tumour size surpassed a volume of 150 mm^3^, mice were stratified based on tumour size to receive daily oral enzalutamide treatment at 60 mg/kg or vehicle control (1% carboxymethylcellulosesodium salt, cat. no. C4888, Sigma-Aldrich with 0.1% Tween 80, cat. no. P1754, Sigma-Aldrich) [11]. We allocated the mice to the different treatment groups based on tumour volumes to minimize the risk of incorporating a bias in baseline tumour volume that could impact treatment response. Once tumours surpassed 300 mm^3^ in size, mice were again stratified based on tumour volume to receive a single intraperitoneal administration of 33 mg/kg cabazitaxel or placebo control (NaCl). Overall, we had four treatment groups, cabazitaxel and enzalutamide mono-treatment, the combination treatment and placebo control, with 9-12 mice each. Tumour volume was monitored every 3-4 days by callipers and mice were followed until tumours exceeded a volume of 1500 mm^3^, or a maximum follow-up of 60 days after cabazitaxel treatment and subsequently euthanized by cervical dislocation. Other reasons for euthanizing the mice included continued weight loss, >15% loss in bodyweight in two days or >20% compared to start, and abnormal behaviour. Mice were maintained in an individually ventilated cage at 2-4 mice per cage, on a 12h dark/light cycle and cage enrichment was provided. Treatments and other procedures were initiated in the morning and food and water were provided ad libitum. Blood samples for PSA analysis were obtained at first tumour measurement, when mice were stratified to receive treatment and at the end of the experiment. For VCaP-Enza-B, we performed a pilot study to assess tumorigenicity, response to enzalutamide and cabazitaxel treatment. Sixteen mice were inoculated with 5*10^6^ VCaP-Enza-B cells mixed with 100 µl Matrigel (cat. no 356231, Corning, NY), tumour take was observed in all mice. Once tumours surpassed 150 mm^3^, mice were stratified based on tumour volume to receive daily enzalutamide treatment (60 mg/kg) or vehicle control for a period of 14 days. Impact of enzalutamide treatment on tumour growth during treatment was assessed using the log_10_-cell kill method [12]. After completing treatment with enzalutamide or placebo control, we exposed nine mice to cabazitaxel for dose optimization. Four mice received an intravenous administration of 33 mg/kg cabazitaxel, three mice received 16 mg/kg and two mice received 10 mg/kg. All mice were followed for at least two weeks to monitor tumour response. Subsequently, we examined the treatment combination of enzalutamide with cabazitaxel versus cabazitaxel alone. Twenty mice were inoculated with 3*10^6^ VCaP-Enza-B cells mixed with Matrigel (cat. no. 356231, Corning, New York), tumour take was observed in 19 out of 20 mice. Once tumours surpassed 150 mm^3^, mice were stratified to receive daily enzalutamide (60 mg/kg) treatment or vehicle control, then all mice received a single intravenous administration of cabazitaxel at tumour volume 750 mm^3^ at 10 mg/kg. Histology of the VCaP-Enza-B tumours and expression of the AR and cell cycle marker Ki67 were subsequently investigated by immunohistochemistry as described previously [7].

### 3.2 RNA-sequencing of enzalutamide treated PC346C-DCC-K tumours

Twenty-one, six-week-old, NMRI nu/nu male mice were subcutaneously inoculated with PC346C-DCC-K cells, tumour take was observed in 20 mice. We estimated that we required 15 samples to perform differential gene expression analysis and therefore transferred five mice to the treatment efficacy experiment described above. The experimental set-up was similar as described above with the following adaptations: mice were stratified to a 2:1 ratio to receive daily enzalutamide treatment or vehicle control for seven days and were subsequently sacrificed and tumour xenografts snap-frozen. Total RNA was isolated from tumour xenografts as described previously [7]. Library Prep was performed using the NEBNext Ultra Directional RNA Kit for Illumina according to the protocol "NEBNext Ultra Directional RNA Library Prep Kit for Illumina" (NEB #E7420S/L). Briefly, mRNA was isolated from total RNA using the oligo-dT magnetic beads. After mRNA fragmentation, cDNA synthesis was performed and used for ligation with adapters and PCR amplification of the resulting product. The quality and yield after sample preparation was measured with the Fragment Analyser. One sample was re-sequenced due high frequency of multi-mapped reads implicating inadequate depletion of ribosomal RNA. Paired-end RNA-Seq data of enzalutamide PDX samples (N = 15) was analysed using UCSC human genome build hg38 and GENCODE annotation release v26 (GRCh38.p10) and mouse genome build mm10 (reference strain C57BL/6J) with GENCODE annotation release M15 (GRCm38.p5) for downstream disambiguation of human/mouse data. FASTQC (v0.11.5) [13] was applied on the paired-end FASTQ files for quality control, both before and after running trimmomatic (v0.36) [14], which removed TrueSeq adapter sequences. STAR (v2.5.3a) was used as aligner, with 2-pass mapping for each sample separately [15]. AstraZeneca's disambiguation algorithm (Python variant, 2013) [16] for reads aligned to two species has been used to assess the best alignments and disambiguate the BAM files. Mapping quality plot was generated and checked based on sambamba Flagstat (v0.6.7) [17] statistics. Count files, with the number of reads for each gene were created with subread FeatureCounts (v1.5.2) [18] on the disambiguated BAM files. R (version 3.4.3) was used for further statistical analyses and data visualization. Differential expression analysis was performed with condition ‘enzalutamide treated’ (N = 10) versus ‘untreated’ (N = 5) using the DESeq2 package (v1.18.1) [19] and the Wald-test. P-values were adjusted using the Benjamini-Hochberg procedure [20]. Settings of different tools can be found in the supplementary data file. Gene set enrichment analysis (GSEA, v4.1.0) was performed using normalized gene expression values (count per million) of the individual tumour samples, with condition ‘enzalutamide treated’ versus ‘untreated’ and applied to the Molecular Signatures Database (MSigDB) hallmark gene set collection [21].

### 3.3 Live cell imaging of cabazitaxel induced microtubule stabilization and apoptosis in PC346C-DCC-K cells

To study the impact of AR pathway inhibition on cellular response to cabazitaxel treatment, PC346C-DCC-K cells were engineered to overexpress enhanced yellow fluorescent protein (EYFP) labelled beta-tubulin (EYFP-β-tubulin) and the human histone *H2B* gene fused to red fluorescent protein (H2B-RFP). The EYFP-β-tubulin expression was used to monitor taxane target engagement and the histone marker for visualizing treatment induced perturbations of mitosis, proliferation and apoptosis. PC346C-DCC-K cells were first transduced to stably express H2B-RFP and selected based on RFP expression. Next cells were transfected with EYFP-β-tubulin and again selected based on fluorescent expression of both markers. Large scale lentiviral production was performed by calcium phosphate transfection of Hek293T (RRID: CVCL_1926) cells with the LV-RFP construct expressing H2B-RFP, pMD2.G plasmid for the viral envelope and psPAX_2_ for packaging [22]. Viral supernatant was collected 24 and 48h after transfection and centrifuged at 3000g to remove cellular debris. The LV-RFP construct expressing H2B-RFP was kindly gifted by Elaine Fuchs (RRID: Addgene_26001, Addgene, Watertown, MA) [23]. Viral supernatant was added to the PC346C-DCC-K cells, incubated overnight and the medium was refreshed the following day. Cells were passaged three times before selection and transfection with the second marker to ensure the viral particles were washed away. For transfection with the expressing EYFP-β-tubulin plasmid (kindly provided by Dr. Galjart, Erasmus MC) we used Lipofectamin 2000 (cat. no. 11668030, Thermo Fisher Scientific, Waltham, Massachusetts, USA) per manufacturer's protocol. Selection PC346C-DCC-K cells was performed by fluorescent activated cell sorting (FACS) using a BD FACS Aria III (BD Biosciences, Franklin Lakes, New Jersey, USA) equipped with 4 lasers and an 85 µm nozzle. mRFP1 fluorescence was detected using a 561 nm yellow-green laser and a 600 LP + 610/20 BP emission filters, similar to mCherry RFP. EYFP fluorescence was detected using a 488 nm blue laser and 502LP + 530/30 BP emission filters, similar to GFP. Dead cells were gated out by means of Hoechst 33258 fluorescence (Hoechst 33258, Sigma Aldrich). Doublets and multicellular clusters were gated out using forward and side scattering according to standard protocols: FSC-W/FSC-A gate, followed by SSC-W/SSC-A gate, verified on FSC-A vs FSC-H gate. Sorting purity, viability, and absence of doublets was verified after sorting, by re-analysis of sorted cells. Cell viability and response to taxane treatment was compared to the parental PC346C-DCC-K, using the MTT assays as described above. No significant impact of the expression of H2B-RFP and EYFP-β-tubulin on taxane sensitivity was found. For live cell imaging, 1*10^4^ PC346C-DCC-K cells co-expressing H2B-RFP and EYFP-β-tubulin were plated with 100 µl PGM-DCC medium in a 96-wells Cell Carrier Ultra plates suited for live cell imaging (cat. no. 6055302, PerkinElmer, Hamburg, Germany). Cells were incubated for 72 hours to ensure optimal attachment and cell spreading. One hour before imaging, cells were pre-treated with 1 µM enzalutamide and/or 0.1 nM R1881. After imaging an initial pre-treatment time point (t_-1_), cells were exposed to 3 nM cabazitaxel and image acquisition was continued for 100 hours with an interval of 150 minutes. During intervals, the cells were stored in an integrated cell culture incubator to optimize cell viability. Sixteen images covering 103714 µm^2^ (containing minimally 1000 cells at the start of the experiment) were acquired using an Opera Phenix spinning-disk confocal high-content screening system (PerkinElmer), equipped with a 40x water immersion object and a 16-bit sCMOS 4 Megapixel camera. EYFP-β-tubulin and H2B-RFP were sequentially excited with 488 nm and 561 nm solid state laser lines, detected at 500-550 nm and 570-630 nm wavelength ranges, respectively. Both analyses, apoptosis induction and microtubule stabilization, were performed using custom image analysis protocols in the Harmony analysis software (version 4.9, Perkin Elmer). First, total area covered by cells was selected using the EYFP-β-tubulin signal. The level of microtubule stabilization was determined in the total area covered by EYFP-β-tubulin expressing cells, using the Haralick Contrast parameter [24,25]. To quantify apoptosis, fragmented nuclei of cells expressing RFP-H2B were detected as spots and clustered by a maximum distance of 5 µm to be assigned to individual apoptotic cells and expressed as percentage to total nuclei.

### 3.4 Statistics

**Figure 1:**
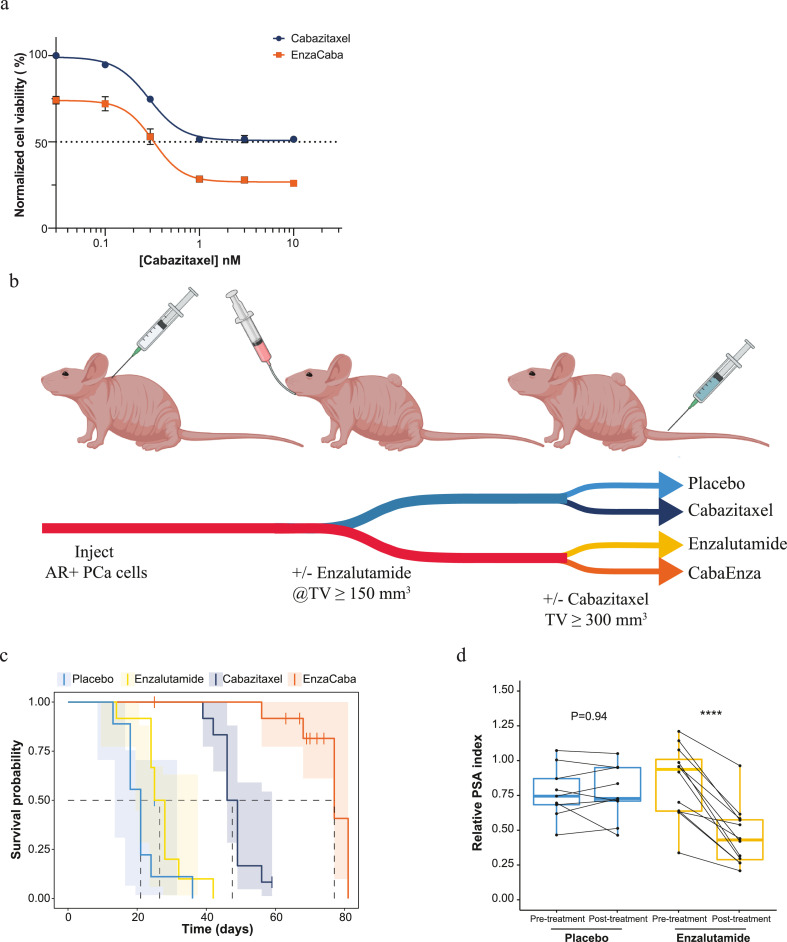
Combined treatment efficacy of enzalutamide and cabazitaxel towards a model of AR positive castrate resistant prostate cancer. a) Efficacy of cabazitaxel and combined with enzalutamide *in vitro* using an AR positive CRPC cell line. Cell viability was assessed by exposing PC346C-DCC-K cells to a dose range of cabazitaxel (blue) alone or with the addition of 1 µM enzalutamide (orange) for 10 days. Shown is the median cell viability of three individual experiments normalized to vehicle controls, error bars display standard error of the mean (SEM) and non-linear regression analysis was used to calculate IC50 values and compared by an extra-sum-of-squares F-test (cabazitaxel IC50 not reached, enzalutamide with cabazitaxel IC50=0.33 nM, P<0.0001). b) Schematic overview of the *in vivo* experimental set-up used to evaluate the combined treatment efficacy of enzalutamide and cabazitaxel, as compared to the respective mono-treatments and placebo. TV = tumour volume, figure made using Biorender. c) Survival analysis of PC346C-DCC-K tumour bearing mice treated with; placebo control (light blue, N=9), enzalutamide (yellow, N=12), cabazitaxel (dark blue, N=12) and the treatment combination (EnzaCaba, orange, N=13). Survival was calculated from the day mice were stratified to start enzalutamide treatment until tumours exceeded a volume of 1500 mm^3^. Mice were censored when tumours did not reach 1500 mm^3^ in size during the maximum follow-up of 60 days after cabazitaxel treatment. One mouse in the EnzaCaba treatment group was found dead two weeks after cabazitaxel treatment. Statistical comparison was performed using a log-rank test with Bonferroni post-hoc analysis. d) Impact of enzalutamide treatment (n=12) on PSA plasma levels as compared to placebo controls (n=9). Pre-treatment plasma samples were collected when mice were stratified to receive daily enzalutamide treatment or vehicle control (placebo). A second, post-treatment, sample was obtained when tumours doubled in size while receiving daily enzalutamide treatment or vehicle control. Y-axis displays relative PSA index which was calculated by dividing PSA levels by tumour volumes at time of sampling. Matched samples are connected by a line, boxplots represent the median values with 25^th^ and 75^th^ percentile and hinges span the 1.5 interquartile range. Statistical comparison was performed by a paired two-sided T-test, **** indicates P<0.0001.

**Figure 2:**
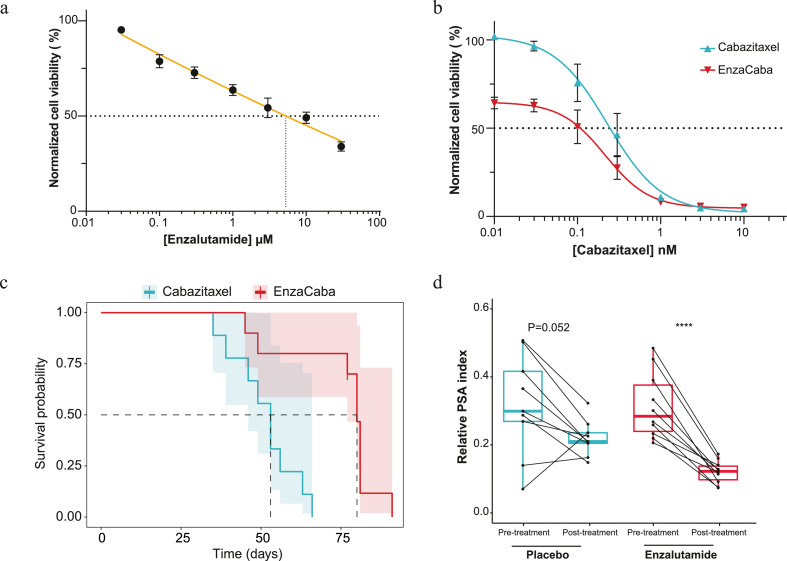
Combined treatment efficacy of enzalutamide and cabazitaxel towards an enzalutamide resistant prostate cancer model. a-b) Efficacy of enzalutamide, cabazitaxel and combination *in vitro* using an AR positive enzalutamide resistant cell line. Cell viability was assessed by exposing VCaP-Enza-B cells to a dose range of enzalutamide (a), cabazitaxel (cyan, b) alone or with the addition of 1 µM enzalutamide (red, b) for 7 days. Shown is the median cell viability of three individual experiments normalized to vehicle controls, error bars display standard error of the mean (SEM) and non-linear regression analysis was used to calculate IC50 values and compared by an extra-sum-of-squares F-test (cabazitaxel IC50=0.24, enzalutamide with cabazitaxel IC50=0.11 nM, P<0.0001) and enzalutamide (IC50 5.4 µM). c) Survival analysis of VCaP-Enza-B tumour bearing mice treated with; cabazitaxel (cyan, N=9) and the treatment combination (EnzaCaba, red, N=10). Survival was calculated from the day mice were stratified to start enzalutamide treatment until tumours exceeded a volume of 1500 mm^3^. Mice were censored when tumours did not reach 1500 mm^3^ in size during the maximum follow-up of 60 days after cabazitaxel treatment. Statistical comparison was performed using a log-rank. d) Impact of enzalutamide treatment (n=10) on PSA plasma levels as compared to placebo controls (n=9). Pre-treatment plasma samples were collected when mice were stratified to receive daily enzalutamide treatment or vehicle control. A second, post-treatment sample, was obtained when tumours exceeded 750 mm^3^ in size while receiving daily enzalutamide treatment or vehicle control. Y-axis displays relative PSA index which was calculated by dividing PSA levels by tumour volumes at time of sampling. Matched samples are connected by a line, boxplots represent the median values with 25^th^ and 75^th^ percentile and hinges span the 1.5 interquartile range. Statistical comparison was performed by a paired two-sided T-test, **** indicates P<0.0001.

The cell viability data obtained from cabazitaxel treated PC346C-DCC-K and VCaP(-Enza-B) cells was assessed using a non-linear regression analysis in GraphPad prism (version 9, GraphPad Software, San Diego, CA). The best-fit values were used to interpolate the IC50 value and compared by an extra-sum-of-squares F-test to determine the impact of the combined treatment with enzalutamide. To determine the impact of cabazitaxel treatment and the combination with enzalutamide *in vivo* we performed a Kaplan Meier analysis in which we defined survival as tumour volumes ≥1500 mm^3^ during the maximum follow-up of 60 days after treatment. Subsequent statistical comparison was performed using a log-rank test with a Bonferroni corrected pairwise comparison, as previously described [7]. Of note, statistical comparison by a non-linear fit model could not accommodate for the average tumour growth dynamics (supplementary figure 1b). We therefore concluded that statistical comparison using median survival outcompeted tumour growth curve analysis. The impact of enzalutamide on PSA plasma levels, as displayed in figure 1d and 2d, were evaluated using a paired two-sided T-test, normality was assessed using Shapiro-test. All tests were performed using the statistical platform R (version 3.6.3). To assess the impact of cabazitaxel treatment and AR pathway inhibition on tubulin stabilization, nuclear fragmentation and proliferation, first, the area under the curve (AUC) and standard errors were calculated. Statistical comparison between the different treatment conditions was subsequently performed by a one-way Welch's Anova analysis with a Dunnett's post-hoc analysis given the non-homogeneity in variance using GraphPad prism.

### 3.5 Role of the funding source

This study was financially supported by an unrestricted grant by Sanofi, however Sanofi was not involved in the design, analysis and interpretation of this study or in writing the manuscript.

## 4 Results

### 4.1 Enzalutamide improves cabazitaxel activity in a model of CRPC

The impact of enzalutamide on cabazitaxel activity was first evaluated in an *in vitro* setting. We used the AR expressing CRPC cell line PC346C-DCC-K, for which we previously reported an interaction between AR signalling and taxane resistance [7]. PC346C-DCC-K harbours intrinsic taxane resistance, as the maximum response to cabazitaxel plateaued at <50% reduction in cell viability (Figure 1a). The addition of 1 µM enzalutamide to the cabazitaxel dose range consistently reduced cell viability by ∼25% compared to cabazitaxel monotherapy (IC50 0·32 nM cabazitaxel, P<0·0001, extra-sum-of-squares F-test). We subsequently investigated the *in vivo* anti-tumour efficacy of combining continuous enzalutamide treatment with a single administration of cabazitaxel, as compared to both mono-treatments and placebo (figure 1b). In line with the *in vitro* results, cabazitaxel was found to be only temporarily effective *in vivo* with 11 out of 12 mice showing initial regression of PC346C-DCC-K tumours followed by rapid outgrowth (Supplementary figure 1a). The combination treatment of enzalutamide and cabazitaxel was able to induce a complete tumour regression in 4/12 mice and substantially delayed the onset of progression in the remaining mice. Of note, the addition of enzalutamide did not result in greater toxicity, as bodyweight loss following cabazitaxel administration was comparable in mice receiving the combination or mono-treatment (Supplementary figure 1b). Overall, combining enzalutamide with cabazitaxel significantly improved tumour response compared to cabazitaxel monotherapy with median time to humane endpoint 77 days and 48 days respectively (P<0·0001, log-rank test with Bonferroni correction, Figure 1c/Supplementary figure 1c). In contrast, enzalutamide monotherapy was found to be ineffective (P=0·17, log-rank test with Bonferroni correction). However, enzalutamide treatment in PC346C-DCC-K tumour bearing mice did lower the PSA plasma levels, suggesting effective inhibition of the AR pathway (P<0·001, T-test, Figure 1d).

### 4.2 Adding enzalutamide to cabazitaxel improves overall activity even in an enzalutamide resistant model

Given the impact of the treatment combination, even in anti-androgen resistant setting, we performed validation studies using an enzalutamide resistant VCaP sub-line. VCaP-Enza-B was selected based on persistent AR signalling activity while being exposed to enzalutamide (Figure 2a, supplementary figure 2). In contrast to PC346C-DCC-K, VCaP-Enza-B displayed high taxane sensitivity, as 1 nM of cabazitaxel reduced cell viability by 90% (Figure 2b). Nevertheless, adding enzalutamide to cabazitaxel further reduced cell viability, as we identified an IC50 shift from 0·24 nM for cabazitaxel to 0·11 nM for the combination (P<0·0001, extra-sum-of-squares F-test). For subsequent *in vivo* studies, we first confirmed enzalutamide resistance of VCaP-Enza-B and optimized cabazitaxel dosing (Supplementary figure 3a-c). Low dose cabazitaxel treatment was found to induce a partial tumour response, thus creating a therapeutic window to study the addition of enzalutamide. Combining enzalutamide with cabazitaxel treatment improved overall activity compared to cabazitaxel monotherapy, with median time to humane endpoint 80 and 53 days respectively (P<0·001, log-rank test with Bonferroni correction, Figure 2c and supplementary figure 3d). Similar to PC346C-DCC-K, enzalutamide treatment reduced PSA plasma levels in VCaP-Enza-B tumour bearing mice while having no impact on tumour growth (Figure 2d). Overall, we conclude that combining enzalutamide and cabazitaxel treatment showed superior anti-tumour efficacy compared to cabazitaxel without targeting AR signalling.

### 4.3 Enzalutamide effectively suppresses AR signalling even in a treatment resistant model

**Figure 3:**
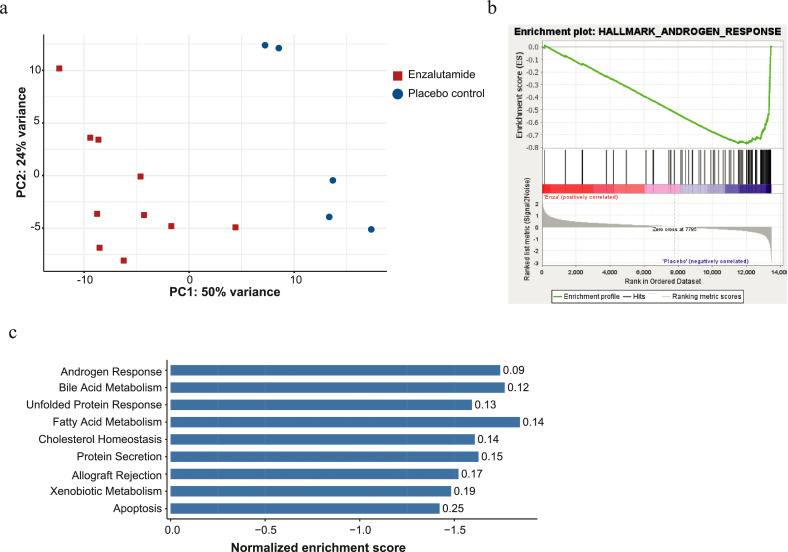
Differential gene expression induced by enzalutamide treatment in PC346C-DCC-K. a) Principal component analysis of RNA expression in PC346C-DCC-K tumours obtained from enzalutamide treated (n=10) and placebo control mice (n=5). Mice were treated for seven days by daily administration of enzalutamide or vehicle control upon which tumours were isolated and used for RNA-sequencing. b-c) Gene set enrichment analysis (GSEA) of differential gene expression caused by enzalutamide treatment in PC346C-DCC-K tumours using the ‘hallmark collection’. b) GSEA plot for the hallmark gene-set ‘Androgen signalling’. c) Overview of significantly enriched gene-sets caused by enzalutamide treatment. Significantly enriched gene-sets were selected based on P value (<0.05) and false discovery rate (FDR <0.25). Y-axis displays the normalized enrichment scores for the individual gene-sets, negative values show pathway inhibition. Gene-sets are ranked based on lowest FDR, thus highest confidence level.

Given the increased efficacy of the combination strategy, we hypothesized that enzalutamide treatment induces gene expression changes that enhance sensitivity to cabazitaxel. In order to identify the pathways that could provoke treatment sensitization, we investigated differential gene expression in short-term enzalutamide treated PC346C-DCC-K tumours using RNA-seq. Although tumour growth of PC346C-DCC-K was unaffected, enzalutamide treatment significantly altered the expression of over 1,900 genes (supplementary data file). Principle component analysis of gene expression data clearly separated tumours obtained from enzalutamide treated from placebo control mice (Figure 3a). Subsequent gene set enrichment analysis (GSEA) identified ‘Androgen response’ as the most significantly altered gene-set, with a normalized enrichment score of -1·74 and a false discovery rate of 0·09 (Figure 3b). Interestingly, several metabolic pathways, including fatty acid metabolism, were significantly repressed by enzalutamide treatment (Figure 3c). GSEA did not identify any significantly upregulated gene-sets that could reflect compensatory growth mechanisms as a result of enzalutamide treatment (Supplementary figure 4). Overall, RNA-sequencing confirmed that enzalutamide treatment repressed AR signalling in PC346C-DCC-K tumours.

### 4.4 Targeting AR signalling enhances cabazitaxel induced apoptosis

**Figure 4:**
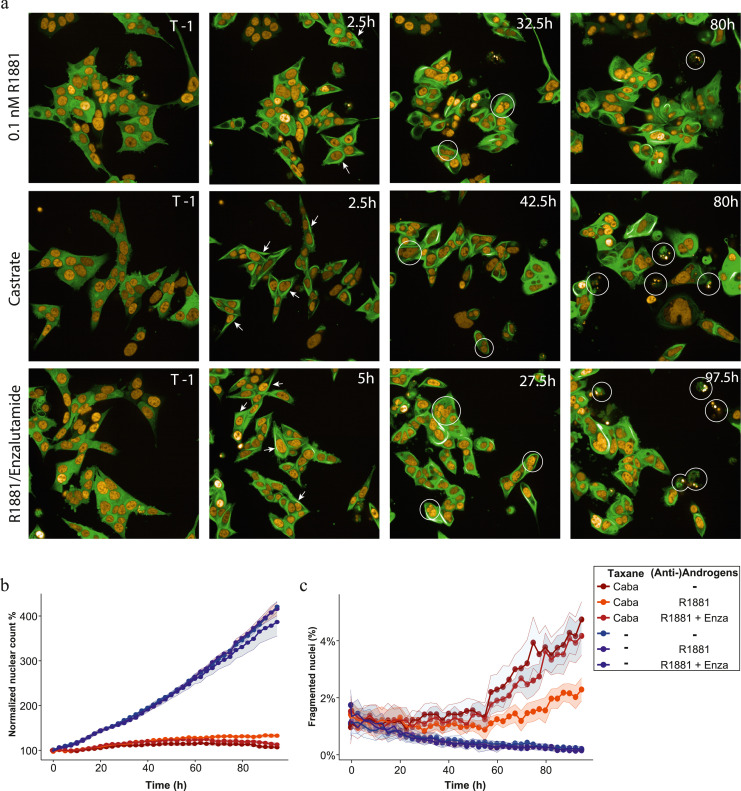
Impact of AR pathway manipulation on cabazitaxel treatment efficacy as studied by live-cell imaging. a) Representative images of PC346C-DCC-K cells expressing EYFP-β-tubulin (green) and H2B-RFP (orange) treated with 3 nM cabazitaxel. The rows represent the different androgen conditions; in the top row culture media was supplemented with 0.1 nM of the synthetic androgen R1881, panels in the middle show cells in castrate culture conditions and bottom show 1 µM of enzalutamide treatment in the presence of R1881. The first column depicts images obtained before addition of cabazitaxel while the second shows images obtained during the first or second interval after taxane treatment. The arrows highlight tubulin structures as a result of cabazitaxel treatment. The third column shows cells treated with cabazitaxel for 24-48h, in which multinucleated cells are circled. The fourth column displays images obtained four days after initiating treatment and highlights fragmented cells which point to treatment induced apoptosis. Scale bars represent 50 µm in size. b-c) Results of image-based quantification of normalized nuclear count (b) and percentage fragmented cells (c). Shown are the results of four individual experiments performed in triplicate, data points represent median values with SD displayed as a band. Nuclear count was used to monitor proliferation and normalized to numbers in pre-treatment images. Fragmented cells were quantified and normalized to nuclear count for each individual time point to calculate the percentage apoptotic cells. Statistical comparison of area under the curve was performed by a one-way Welch's Anova with Dunnett's post-hoc analysis.

Taxane chemotherapeutics exert their function by targeting tubulins leading to microtubule stabilization and cell cycle stalling, which can trigger cell death by mitotic catastrophe. To further unravel the impact of AR signalling on cabazitaxel efficacy, we used live-cell imaging. PC346C-DCC-K cells were transfected to dually express fluorescently labelled H2B (Figure 4a, orange nuclear staining) and β-tubulin (green cytoplasmic staining). We used these markers to track taxane induced perturbations of mitosis and tubulin dynamics respectively. After cabazitaxel exposure (3 nM) we observed rapid formation of tubulin bundles with high fluorescent intensity, indicative of taxane induced tubulin stabilization (Figure 4a, second column). As expected, tubulin stabilization induced abnormal and prolonged mitosis, which led to mitotic slippage and multinucleated cells (Figure 4a, third column). In androgen supplemented conditions (R1881), treatment induced apoptosis was rarely observed. At first, we did not observe a substantial impact of either castrate (minus R1881) or enzalutamide treated conditions on cabazitaxel efficacy. However, after >48h of taxane exposure in AR suppressed conditions, secondary cell divisions more frequently resulted in apoptosis. To validate these observations, we used image-based quantification of proliferation, apoptosis and tubulin stabilization. We confirmed that cabazitaxel treatment induced rapid tubulin stabilization as identified by Haralick contrast (Supplementary figure 5a). Although proliferation was suppressed by cabazitaxel treatment, androgen stimulation induced a significant increase in nuclear count (P<0·0001, one-way Welch's Anova with Dunnett's post-hoc, Figure 4b, Supplementary figure 5b,). The low percentage of fragmented nuclei caused by apoptosis (1.5-2.5%) confirmed our observation of cabazitaxel resistance in androgen supplemented conditions (Figure 4c). Either castrate culture conditions or the addition of enzalutamide increased the percentage of cabazitaxel induced apoptotic cells by 1·5-2% (P<0·001, one-way Welch's Anova with Dunnett's post-hoc).

## 5 Discussion

The present study explored whether cabazitaxel treatment efficacy could be enhanced by targeting the AR. In two PDX models we showed that adding enzalutamide to cabazitaxel induced greater anti-tumour activity, compared to both single treatments. Importantly, the combination treatment was found to remain active in enzalutamide resistant CRPC models where AR downstream signalling was significantly impacted by enzalutamide treatment, although failed to impact tumour growth by itself. Importantly, targeting the AR increased the rate of cabazitaxel induced apoptosis. Overall, our study shows that cabazitaxel efficacy is directly affected by AR signalling, which paves the way for promising combination treatments in CRPC.

Taxane chemotherapeutics exert their function by targeting the β-tubulin subunit of the tubulin polymer, which is part of the microtubule network, and induces stabilization. During mitosis, taxane treatment interferes with chromatid separation and eventually leads to cell cycle stalling in the G2/M phase. Arrest in mitosis can directly induce cell death by mitotic catastrophe, although cancer cell lines have shown to display variable response to taxane treatment [26]. Live-cell imaging revealed that in taxane resistant PC346C-DCC-K cells, cabazitaxel treatment effectively induced cell cycle stalling, mitotic slippage and multinucleation, although this rarely triggered mitotic cell death. Adding enzalutamide further suppressed proliferation and increased the percentage of fragmented cells, but only after 48h of taxane treatment. Targeting the AR pathway therefore likely decreased cell viability after the first abnormal cell cycle. Although, the impact of enzalutamide co-administration increased the percentage of apoptotic cells by only 1·5-2%, we reasoned that the continued cell cycle perturbations over time will result in a consistent accumulation of apoptotic cells, which will significantly contribute to the therapeutic efficacy as so clearly observed *in vivo*.

Sustained AR signalling plays a major role in CRPC, as exemplified by the frequency of genetic aberrations and splice-variant expression driving resistance to AR targeted treatment [3,27]. The current study, together with our previous reports, implicates a major role for AR signalling in taxane treatment efficacy [6,7]. Of note, the impact of AR signalling on taxane treatment efficacy seemed to be magnified in the *in vivo* setting, as the *in vitro* cell viability assays showed an additive interaction of enzalutamide combined with cabazitaxel. Overall, the underlying mechanisms resulting in greater cabazitaxel sensitivity in enzalutamide treated PCa cells remain to be clarified, although several metabolic pathways were significantly impacted by enzalutamide treatment. Potentially, multinucleated cells may become sensitive to enzalutamide induced suppression of metabolic activity leading to increased rates of apoptosis. Clearly further investigation is warranted to unravel if these pathways contribute to the enhanced cabazitaxel responsiveness.

A preclinical study by Martin et al., also reported an added benefit of combining cabazitaxel with AR signalling targeted treatment in AR positive PCa models [28]. The authors concluded that adding enzalutamide to cabazitaxel was effective in hormone sensitive PCa but not in CRPC cell lines. Moreover, the addition of enzalutamide to cabazitaxel did not significantly decrease the tumour mass of 22Rv1 xenografts. However, activity of the combination treatment was assessed using a different dosing schedule and based on a single tumour measurement obtained four days after completing the cabazitaxel treatment. In our *in vivo* experiments, the greatest impact of the combination treatment was on long-term suppression of tumour-growth after cabazitaxel treatment. Martin et al. did identify an impact of the combination treatment on the frequency of proliferating and apoptotic cells upon further inspection of the 22Rv1 tumours. Overall, we argue that a premature endpoint potentially compromised the assessment of the combination treatment.

The addition of enzalutamide to cabazitaxel in CRPC patients, has been investigated in a phase I clinical trial performed at our institute [29]. Enzalutamide is a known CYP3A4 inducer and could impact on cabazitaxel exposure through increased clearance. Indeed, concomitant enzalutamide treatment was found to reduce cabazitaxel plasma levels by 22%. We have previously shown that adequate taxane exposure is key for optimal anti-tumour activity [30]. However, in the clinical study two cycles of the combination treatment was still found to induce a >50% PSA reduction in 8/13 CRPC patients [29]. This suggests that combining enzalutamide with cabazitaxel is an effective treatment, despite the moderately decreased systemic cabazitaxel exposure. Moreover, our current preclinical data suggest that AR positive patients who are no longer responsive to enzalutamide treatment, could still benefit from combining enzalutamide with cabazitaxel. Treatment resistant PCa who display neuro-endocrine features, including loss of AR pathway activity, are unlikely to benefit from the addition of enzalutamide to cabazitaxel. Several ongoing phase II/III clinical trials investigate the feasibility of combining AR signalling targeted agents with taxane chemotherapeutics for advanced PCa [31]. With the treatment landscape of mPCa moving towards effective combination strategies, adding AR signalling targeted inhibitors to taxane chemotherapeutics provides a promising strategy for CRPC patients.

## 6 Contributors

The manuscript was written by LM, RM, RdW, ML, and RdW and reviewed by all authors. The study was designed by LM, RdW, ML and WvW. The VCaP-Enza-B cell line was obtained and characterized by RM, the PC346C-DCC-K H2B/tubulin expressing cell line was obtained and characterized by LM. In vitro experiments were performed by LM and SB. All animal experiment were designed by LM and WvW and performed by CdR and DS. The RNA-sequencing experiment was designed by LM, ML and HvdW and analysed by WvdG and LM. Experimental set-up and data analysis of live-cell imaging experiment was performed by LM and MvR.

## Declaration of Competing Interest

RDW—Advisory role/speaker fees; Sanofi, Merck, Lilly, Roche, Bayer, Janssen, Clovis and research funding (Institutional); Sanofi, Bayer. MPL—Advisory role/speaker fees; Incyte, Amgen, Janssen Cilag B.V., Bayer, Servier, Roche, Pfizer Sanofi Aventis Netherlands BV, Astellas and has received research funding (Institutional) from Sanofi, JnJ, Merck and Astellas. RHJM—has received research funding (Institutional) from Sanofi and Astellas. WvW—has received research funding (Institutional) from Sanofi, Millennium, Janssen Pharmaceuticals, and Servier. LM—Advisory role/speaker fees from Sanofi. HvdW — Advisory role/speaker fees from Bayer.

## Appendix Supplementary materials

Image, application 1

Image, application 2

Image, application 3

Image, application 4
