# Supplementary material for: Continued Androgen Signalling Inhibition improves Cabazitaxel Efficacy in Prostate Cancer
Source: eBioMedicine. 2021 Nov 5;73:103681. doi: 10.1016/j.ebiom.2021.103681 (PMC8586743; doi:10.1016/j.ebiom.2021.103681)
Supplement: Supplementary file 2 [file mmc2.docx]

Caption for supplementary material:

Supplementary figures 1-5

Supplementary figure legends 1-5

Supplementary data file RNA-seq
