# Supplementary material for: Continued Androgen Signalling Inhibition improves Cabazitaxel Efficacy in Prostate Cancer
Source: eBioMedicine. 2021 Nov 5;73:103681. doi: 10.1016/j.ebiom.2021.103681 (PMC8586743; doi:10.1016/j.ebiom.2021.103681)

Supplementary figure 1

a

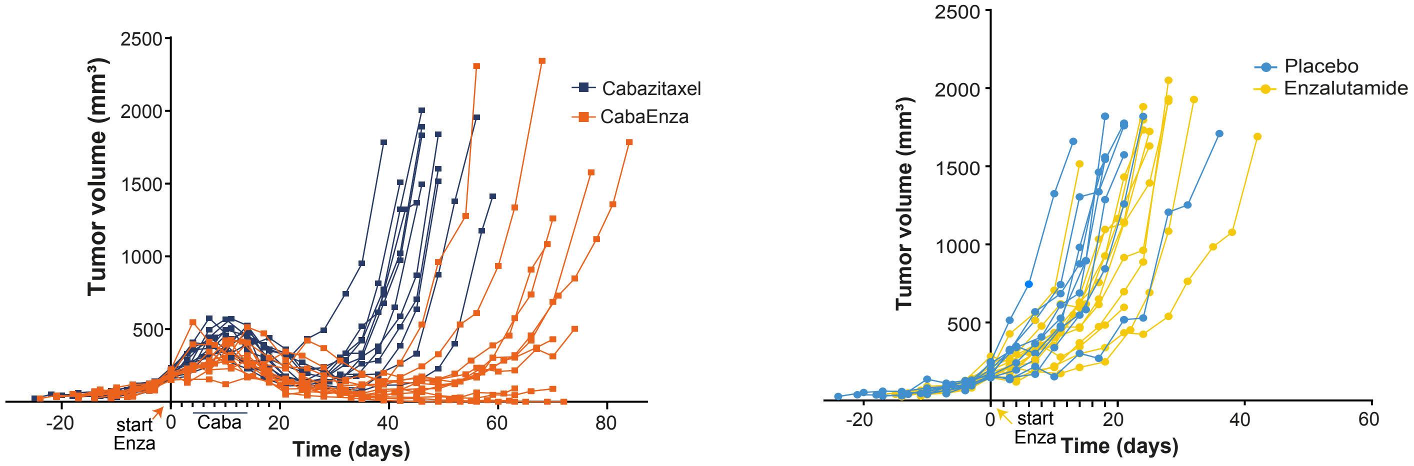

b

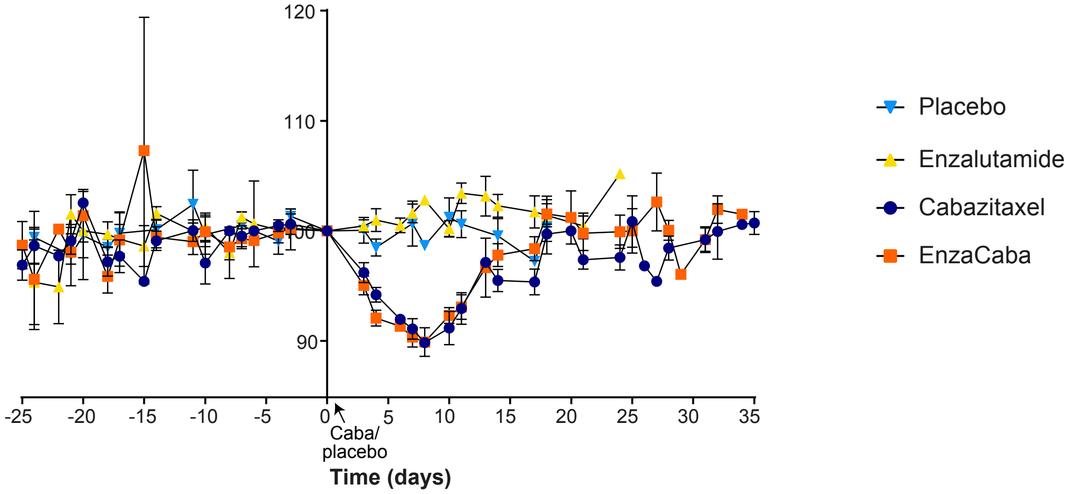

c

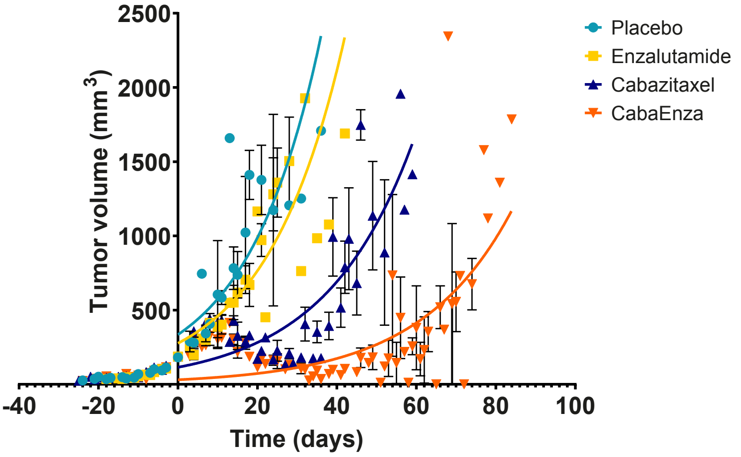

d

	EnzaCaba	Cabazitaxel	Enzalutamide
Cabazitaxel	$8.8*10^{-5}$		
Enzalutamide	$3.3*10^{-6}$	$2.0*10^{-5}$	
Placebo	$2.4*10^{-6}$	$5.3*10^{-6}$	0.17

Supplementary figure 2

a

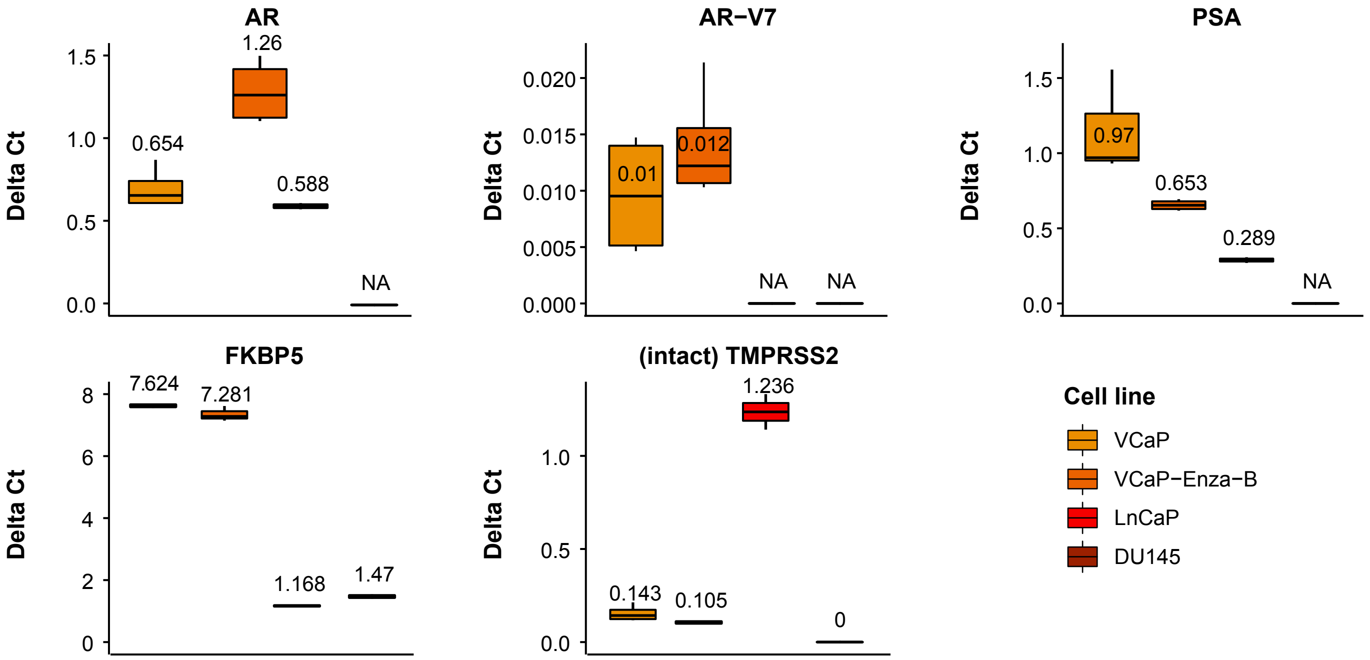

b

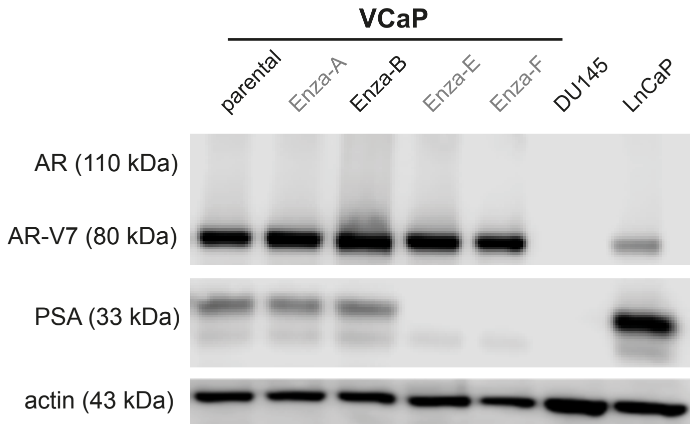

c

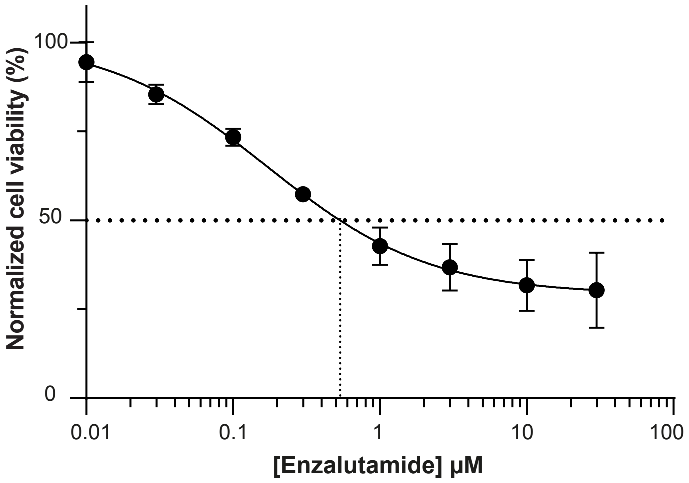

Supplementary figure 3

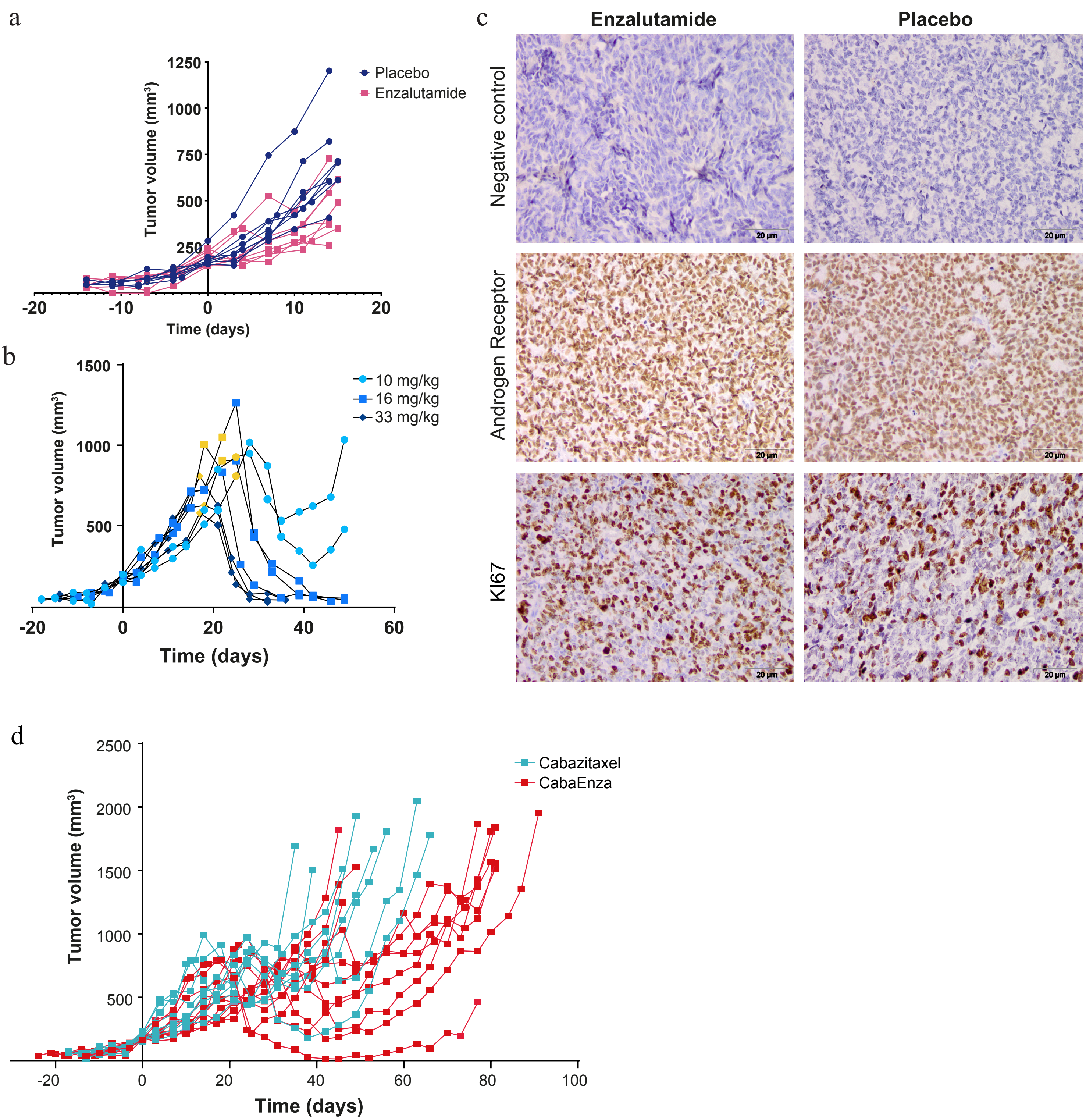

Supplementary figure 4

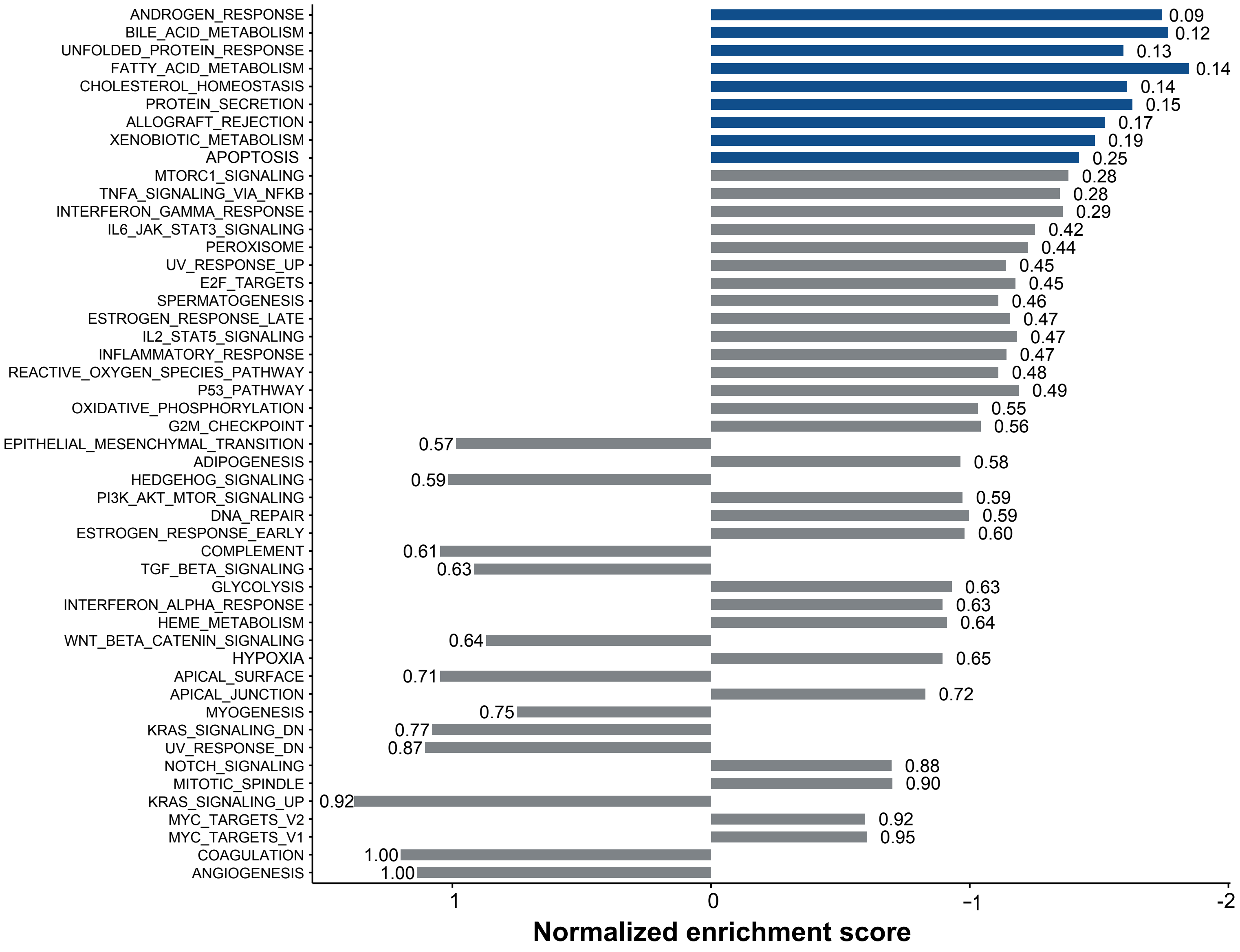

Supplementary figure 5

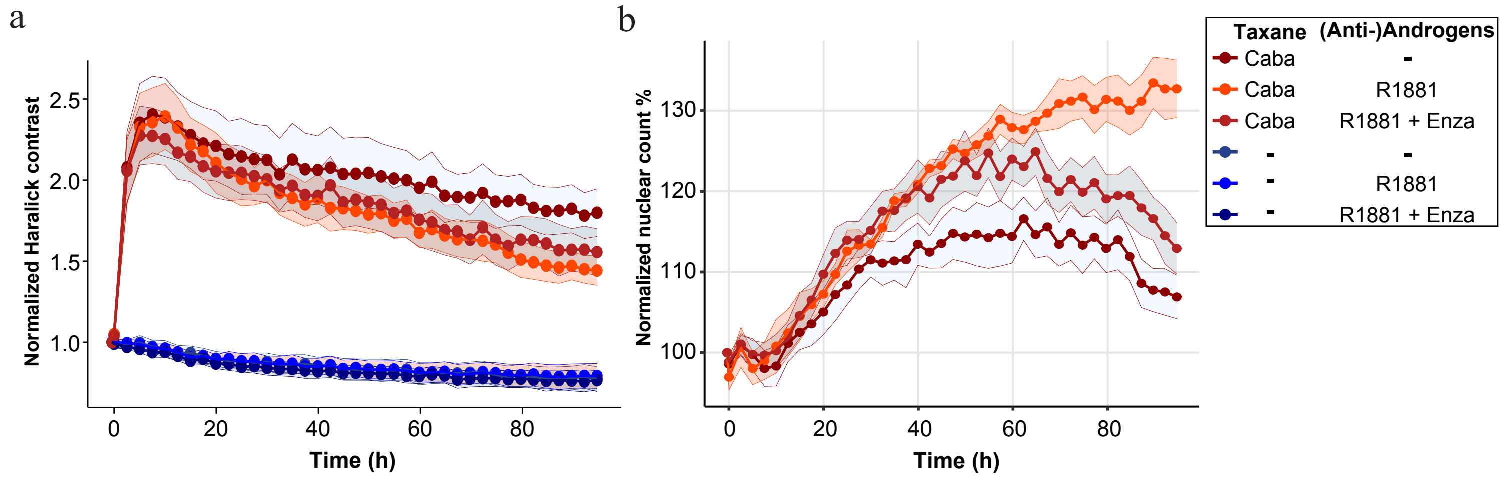

Supplement: Supplementary file 3 [file mmc3.pdf]
