# Supplementary material for: Continued Androgen Signalling Inhibition improves Cabazitaxel Efficacy in Prostate Cancer
Source: eBioMedicine. 2021 Nov 5;73:103681. doi: 10.1016/j.ebiom.2021.103681 (PMC8586743; doi:10.1016/j.ebiom.2021.103681)
Supplement: Supplementary file 4 [file mmc4.docx]

**Supplementary figure legends**

**Supplementary figure 1: *In vivo* anti-tumour efficacy of the enzalutamide and cabazitaxel combination treatment towards the PC346C-DCC-K patient derived xenograft. a)** Individual PC346C-DCC-K tumour growth curves. Mice were treated with cabazitaxel with or without enzalutamide (left panel) or placebo control versus enzalutamide (right panel). Day of treatment initiation for enzalutamide (enza) and cabazitaxel (caba) is indicated on the graph, enzalutamide was administered daily. **b)** Normalized bodyweights of tumour bearing mice receiving cabazitaxel (dark blue), enzalutamide (yellow) or combination treatment (orange) compared to placebo controls (light blue). Bodyweights of individual mice were normalized to weight on day 0, when mice were stratified to receive cabazitaxel treatment or placebo control (indicated on the graph as “Caba/placebo”). Displayed are the median normalized bodyweights (%) per treatment group with error bars showing standard error of the mean (SEM). Loss of bodyweight induced by cabazitaxel treatment did not surpass 20% which is defined as a humane endpoint. **c)** Average PC346C-DCC-K tumour growth curves. Datapoints represent mean tumour growth +/- SEM, a non-linear fit model was used to assess treatment response although this resulted in poor fit values (R^2^: 0.22-0.5). **d)** Table displaying the outcome of Bonferroni corrected multiple comparison of log-rank survival analysis shown in Figure 1c.

**Supplementary figure 2: characterization of enzalutamide resistant VCaP cell line. a-b)** Characterization of the AR pathway in the VCaP and VCaP-Enza-B clone. Cell were exposed to standard culture conditions at time of isolation, which includes 10 µM enzalutamide for the VCaP-Enza-B clone. **a)** RNA expression of the androgen receptor (*AR*), AR-variant 7 (*AR-V7*) and the target genes *FKBP5*, *KLK3* and *TMPRSS2* as assessed by qRT-PCR. The hinges of the boxplots represent the 25^th^ and 75th percentile with median expression and the whiskers represent 1.5x the interquartile range (IQR). For each gene 2-4 technical replicates were included, and expression was normalized to two household genes using the 2^-ΔCt^ method. NA indicated target genes not expressed by the cell line. **b)** Protein expression of AR, AR-V7 and PSA as assessed by western blot. Other enzalutamide resistant VCaP clones, not used for this study, were included in this blot. Protein lysate from the DU145 cell line was used as a negative control for AR, AR-V7 and PSA expression while LNCaP was used as a positive control for AR and PSA expression. Actin was used as a loading control. **c)** Enzalutamide sensitivity of the parental VCaP cell line. Shown is the median cell viability of two individual experiments normalized to vehicle controls, error bars display standard error of the mean (SEM) and non-linear regression analysis was used to calculate IC50 value (0.54 µM).

**Supplementary figure 3: *In vivo* characterization and treatment sensitivity of VCaP-Enza-B. a)** Enzalutamide resistance of the VCaP-Enza-B tumour model. Shown are individual tumour growth curves of mice receiving either daily enzalutamide treatment or placebo control for two weeks. Impact of enzalutamide treatment was assessed using the log_10_-cell kill calculation (0.087), which confirmed that VCaP-Enza-B was enzalutamide resistant (log-cell kill <0.7). **b)** Response of the VCaP-Enza-B tumour model to different cabazitaxel dosages. Subset of VCaP-Enza-B tumour bearing mice as displayed in A were subjected to either 10 mg/kg (N=2 light blue), 16 mg/kg (N=3, blue) or 33 mg/kg (N=4, dark blue) cabazitaxel treatment. Timing of cabazitaxel administration is highlighted in yellow. **c)** Histological assessment and immunohistochemical staining of the androgen receptor and Ki67 in VCaP-Enza-B tumours. **d)** Individual VCaP-Enza-B tumour growth curves of mice treated with cabazitaxel with or without enzalutamide.

**Supplementary figure 4: Gene set enrichment analysis of differential gene expression induced by enzalutamide treatment in PC346C-DCC-K tumours.** Gene set enrichment analysis (GSEA) using the Hallmarks gene sets applied to differential gene expression induced by enzalutamide treatment in PC346C-DCC-K tumours. Shown is an overview of normalized enrichment scores (Y-axis) of the individual gene sets and the matching false discovery rate (FDR). Gene sets that meet the significance, with a FDR ≤0.25, are highlighted in blue.

**Supplementary figure 5: Impact of AR pathway manipulation on cabazitaxel treatment efficacy. a-b)** Results of image-based quantification of Haralick contrast (a) and normalized nuclear count of cabazitaxel treated samples (b). Shown are the results of four individual experiments performed in triplicate, data points represent median values with SD displayed as a band. Haralick contrast measures pixel to pixel differences in fluorescent intensities and was applied to inspect tubulin structures. Normalization to Haralick contrast levels in pre-treatment images was performed. The slow reduction in Haralick contrast over time is most likely a result of monitor bleaching. Nuclear count was used to monitor proliferation and normalized to numbers in pre-treatment images. Statistical comparison of area under the curve was performed by a one-way Welch’s Anova with Dunnett’s post-hoc analysis.
